# β-Cyclodextrin Inclusion Complexes of Curcumin and Synthetic Analogues in PVA/Carrageenan Hydrogels: A Platform for Sustained Release and Microbial Control

**DOI:** 10.3390/gels11110922

**Published:** 2025-11-18

**Authors:** Patricia Daiane Zank, Matheus da Silva Gularte, André Ricardo Fajardo, Matheus Pereira de Albuquerque, Vithor Parada Garcia, Rafaely Piccioni Rosado, Letícia Zibetti, Clarissa Piccinin Frizzo, Bruno Nunes da Rosa, Cláudio Martin Pereira de Pereira, Janice Luehring Giongo, Rodrigo de Almeida Vaucher

**Affiliations:** 1 Biology Research Laboratory, (LAPEBBIOM), Department of Chemical, Pharmaceutical, and Food Sciences, Microorganism Biochemistry and Molecular, Federal University of Pelotas, Pelotas 96010-610, RS, Brazil; 2 Laboratory of Technology and Development of Composites and Polymeric Materials (LaCoPol), Federal University of Pelotas, Pelotas 96010-900, RS, Brazil; 3 Heterocycle Chemistry Nucleus (NUQUIMHE), Federal University of Santa Maria, Santa Maria 97105-340, RS, Brazil; 4 Lipidomics and Bioorganic Laboratory, Center for Chemical, Pharmaceutical and Food Sciences, Federal University of Pelotas, Pelotas 96010-900, RS, Brazil; 5 Faculty of Medicine (FAMED), Pharmacy of Course, Federal University of Rio Grande, Rio Grande 92203-900, RS, Brazil

**Keywords:** vaginal infections, *Gardnerella vaginalis*, antimicrobial activity, women’s health, drug delivery systems

## Abstract

This study describes the development of β-cyclodextrin (β-CD) inclusion complexes of curcumin (CUR) and a synthetic curcuminoid analogue (CN56), which were incorporated into poly(vinyl alcohol)/κ-carrageenan hydrogel films to create a multifunctional system capable of sustained drug release and effective antimicrobial action. Carrageenan was extracted from *Gigartina skottsbergii*, and hydrogels were prepared using a freeze–thaw crosslinking method. The inclusion complexes were formed at a 1:6 molar ratio, achieving loading efficiencies of 75.62% for CUR and 79.00% for CN56. FTIR confirmed molecular interactions between the complexes and the polymeric matrix, accompanied by reduced crystallinity and increased amorphous character. Thermogravimetric analysis revealed enhanced thermal stability, with degradation onset temperatures above 239 °C, while DSC analysis indicated irreversible amorphization after the first heating cycle. SEM analysis showed improved surface uniformity in complex-loaded films compared with those containing free compounds. Swelling experiments demonstrated significantly greater fluid uptake in complex-loaded hydrogels, particularly for CN56 (1080% after 45 min). Controlled release studies revealed sustained drug release profiles, with 76.49% of CUR and 56.02% of CN56 released over 36 h, following Fickian diffusion mechanisms. In vitro antimicrobial assays confirmed marked activity of CUR and CN56 against *Gardnerella vaginalis*, a key pathogen associated with bacterial vaginosis. Biocompatibility tests, including hemolysis and MTT reduction assays, indicated low cytotoxicity and satisfactory hemocompatibility. Rheological analysis further demonstrated increased viscosity and potential mucoadhesive behavior. Collectively, these findings highlight the potential of carrageenan-based PVA hydrogels as innovative pharmaceutical platforms for the prevention and treatment of recurrent bacterial vaginosis, offering a promising alternative to conventional therapies.

## 1. Introduction

The search for natural bioactive compounds with multifunctional properties has attracted growing attention from both the scientific and industrial communities, particularly for applications in the pharmaceutical, cosmetic, and biomedical fields. Among natural polysaccharides, carrageenans—sulfated galactans extracted from red seaweeds (*Rhodophyta*)—have demonstrated diverse biological activities, including antioxidant, antimicrobial, antiviral, and immunomodulatory effects [1,2,3,4]. These biopolymers also exhibit favorable physicochemical characteristics, such as high viscosity, gelling ability, and excellent biocompatibility, making them promising candidates for the development of drug delivery systems and topical formulations [5].

The red macroalga *Gigartina skottsbergii* is notably rich in κ/λ-carrageenan [6] and has been extensively studied for its antiviral and immunomodulatory properties, particularly against herpes simplex viruses [7]. However, despite its ecological and commercial importance in southern Chile and the Antarctic Peninsula, the biotechnological potential of carrageenan derived from *G. skottsbergii* remains underexplored—especially regarding its suitability as a biomaterial for targeted therapeutic applications.

One of the most promising frontiers for biomaterial innovation lies in women’s health. Vaginal infections such as bacterial vaginosis (BV) and vulvovaginal candidiasis affect millions of women worldwide, with BV alone accounting for nearly 30% of cases among women of reproductive age [8,9]. The condition is strongly associated with *Gardnerella vaginalis*, which plays a central role in biofilm formation and disruption of the vaginal microbiota [10]. Current pharmacological therapies, including antibiotics and antifungals, often result in high recurrence rates and the emergence of resistant strains, underscoring the urgent need for alternative therapeutic strategies [11,12].

Hydrogels have emerged as promising drug delivery systems for vaginal applications due to their ability to adhere to mucosal tissues, prolong residence time, and provide controlled release of bioactive compounds [13,14]. Recent reviews have highlighted the development of antimicrobial polymer-based hydrogels for intravaginal therapies, emphasizing mechanisms of bioadhesion, thermoresponsive behavior, and improved solubility of hydrophobic drugs [15]. Within this context, carrageenan-based systems have gained prominence as multifunctional biomaterials for pharmaceutical and biomedical applications, including tissue engineering, wound healing, and drug delivery [16,17]. Furthermore, combinations of κ-carrageenan and poly(vinyl alcohol) (PVA) have been shown to yield hydrogels with favorable mechanical strength, swelling behavior, and antimicrobial performance for biomedical use [18,19,20]. PVA, a biocompatible and water-soluble synthetic polymer, is widely utilized in biomedical formulations due to its film-forming capacity, flexibility, and ability to enhance the structural stability of natural polysaccharide matrices. Nevertheless, most of these formulations do not incorporate molecular inclusion systems to improve the stability or bioavailability of hydrophobic bioactives—an enduring challenge for efficient delivery applications.

Cyclodextrin-based inclusion complexes have recently emerged as powerful tools to enhance the solubility, stability, and controlled release of hydrophobic molecules such as curcumin and synthetic curcuminoids [21]. When incorporated into polymeric matrices like PVA and carrageenan, these complexes can promote structural reorganization, influence swelling dynamics, and modulate drug release kinetics [5]. Additionally, the freeze–thaw crosslinking technique provides a mild, solvent-free approach to fabricate physically crosslinked hydrogels with defined porosity and mechanical robustness suitable for biomedical applications [22].

This study aims to develop and characterize carrageenan/PVA hydrogel films incorporating curcumin and a synthetic curcuminoid through β-cyclodextrin inclusion complexes. The physicochemical, thermal, and morphological properties of the films were evaluated alongside in vitro antimicrobial assays against *Gardnerella vaginalis* and *Candida albicans*. By emphasizing the biological and technological relevance of these formulations, this work proposes carrageenan-based hydrogels as potential pharmaceutical platforms for the prevention and treatment of vaginal infections. The novelty of this study lies in combining Antarctic-derived *Gigartina skottsbergii* carrageenan with β-cyclodextrin-complexed curcuminoids within PVA hydrogels—an unexplored approach designed to enhance bioactive stability and antimicrobial efficacy against key pathogens affecting women’s health.

## 2. Results and Discussion

### 2.1. Formation of β-CD Inclusion Complexes

The supramolecular structure of β-cyclodextrin (β-CD) contains a hydrophobic inner cavity capable of accommodating curcumin (CUR), forming an inclusion complex that enhances its solubility in aqueous environments [23]. In this study, β-CD inclusion complexes were prepared by adding CUR or the synthetic curcuminoid CN56 to an aqueous β-CD solution at a molar ratio of 1:6. According to the literature, this ratio is considered optimal for complete encapsulation of CUR, since the molecular dimensions of CUR (approximately 19 Å in length and 6 Å in width) exceed the internal diameter of β-CD (~7.8 Å), thereby requiring the participation of two or more β-CD units to accommodate each phenolic ring. This supramolecular arrangement promotes the formation of more stable and efficient complexes. As a control, CUR was also subjected to the same procedure in the absence of β-CD [24].

The formation of the inclusion complexes was monitored by UV–Vis spectroscopy. In the presence of β-CD, a progressive decrease in the characteristic absorption band near 425 nm was observed, indicating the gradual incorporation of CUR into the hydrophobic cavity of β-CD. In contrast, no spectral change occurred in the control sample without β-CD, confirming the chemical stability of CUR under the experimental conditions (pH 5). At this pH, CUR predominantly exists in its β-keto–enol tautomeric form, which favors intra- and intermolecular hydrogen bonding, thereby promoting molecular crystallization. Upon complexation with β-CD, these intermolecular interactions are disrupted, as the inclusion process displaces high-energy water molecules from the β-CD cavity, increasing system entropy and rendering the process thermodynamically favorable [25].

The efficiency of complex formation was evaluated by determining the loading efficiency (LE), which was approximately 75.62% for CUR and 79.00% for CN56. These results confirm the effectiveness of the encapsulation procedure and are consistent with previous reports suggesting that, in addition to molecular inclusion, mechanisms such as micellar aggregation may also contribute to the stabilization of these complexes [26].

In summary, the use of a 1:6 molar ratio proved crucial for obtaining stable β-CD inclusion complexes with enhanced solubility and bioavailability of CUR and CN56—key prerequisites for the development of controlled vaginal drug delivery systems.

### 2.2. FTIR Analysis

**Figure 1:**
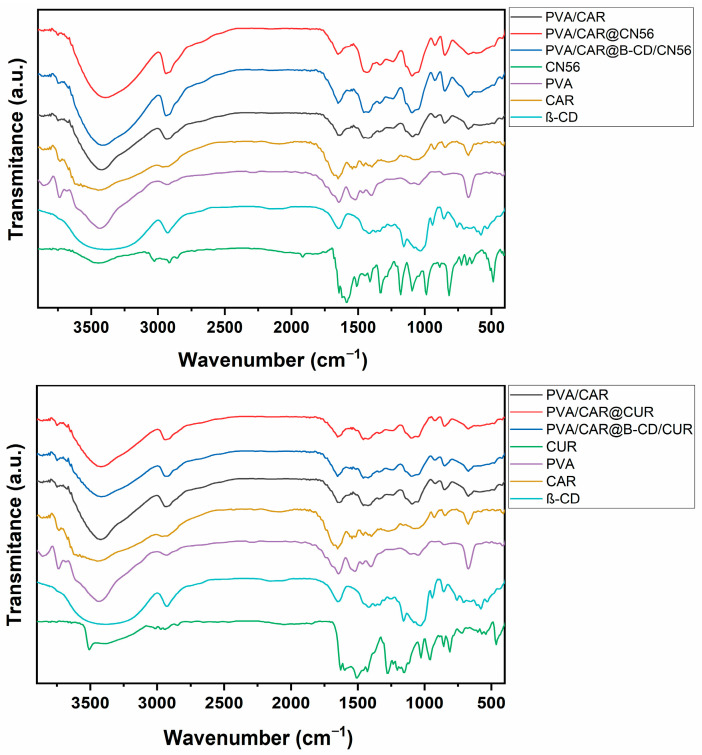
FTIR spectra of pure components, free and complexed CUR and CN56, and their respective PVA/CAR-based films.

The chemical interactions between the β-cyclodextrin (β-CD) inclusion complexes and the poly(vinyl alcohol)/carrageenan (PVA/CAR) polymeric matrix were evaluated by Fourier-transform infrared (FTIR) spectroscopy (Figure 1). The FTIR spectrum of the pure PVA/CAR film exhibited characteristic absorption bands of both polymers, including a broad O–H stretching band at 3425 cm^−1^, C–H stretching bands between 2989 and 2800 cm^−1^, a water-associated bending band at 1632 cm^−1^, and a C–O stretching band at 1543 cm^−1^ attributed to acetyl residues in PVA [13]. In addition, carrageenan-specific peaks were observed at 847, 926, and 1037 cm^−1^—corresponding to C–O–SO_3_^−^ vibrations, CH_2_ rocking of 3,6-anhydro-D-galactose, and glycosidic C–O–C stretching, respectively—as well as at 1159 and 1262 cm^−1^, which are characteristic of sulfate-ester S=O stretching [27].

The spectra of the films containing β-CD/CUR and β-CD/CN56 complexes displayed overall similarity to that of the pure matrix but exhibited subtle spectral shifts, confirming the successful incorporation of the inclusion complexes. A slight narrowing of the broad O–H stretching band was observed, accompanied by downshifts in the deformation bands associated with O–H and C–OH groups, now appearing at 1334 cm^−1^ and 1234 cm^−1^, respectively. Moreover, an intensified absorption band at approximately 840 cm^−1^ was detected, which may be attributed to interactions between carrageenan sulfate groups and the β-CD inclusion complexes [3]. These spectral modifications collectively indicate the formation of new hydrogen-bonding interactions and strong matrix–complex associations during film formation [24], supporting the successful integration of β-CD/CUR and β-CD/CN56 complexes within the PVA/CAR hydrogel network.

In the fingerprint region (1200–1000 cm^−1^), noticeable variations were observed in the relative intensities of the bands at 1143 cm^−1^ and 1098 cm^−1^, corresponding to C–O stretching vibrations associated with crystalline and amorphous domains, respectively. The intensity ratio of these bands (I_1143_/I_1098_) is commonly employed to estimate the degree of crystallinity in PVA-based systems prepared via freeze–thaw processing [24]. In the present study, a decrease in this ratio was detected for films containing the inclusion complexes, indicating a reduction in crystallinity and an increase in amorphous character. This finding aligns with previous reports showing that the incorporation of hydrophobic bioactive molecules or cyclodextrin complexes can disrupt the ordered packing of PVA chains [5].

Collectively, the FTIR results confirmed the successful incorporation of β-CD/CUR and β-CD/CN56 complexes within the polymeric matrix and supported the hypothesis that these complexes interact with the functional groups of PVA and carrageenan, modifying the supramolecular organization. Such structural alterations may contribute to the observed differences in film swelling and drug-release behavior [5]. These interactions suggest good compatibility among the polymeric components; however, they do not by themselves establish mechanical stability or performance under physiological conditions. Therefore, additional rheological and texture analyses are required to evaluate mechanical strength, spreadability, and retention properties relevant to vaginal applications.

### 2.3. Thermogravimetric Analysis (TGA)

Thermogravimetric analysis (TGA) revealed clear distinctions between the thermal behavior of pure carrageenan and that of the developed biofilms. A summary of all TGA parameters is provided in Table 1. The pure carrageenan sample exhibited an initial mass loss of approximately 5% (Td_5_%) at 56 °C, which can be attributed to the evaporation of physically adsorbed water or residual volatile compounds. Thermal degradation occurred in two main stages, with maximum decomposition temperatures (Td_1_ and Td_2_) at 167 °C and 185 °C, respectively, resulting in an overall mass loss of 42% within the analyzed temperature range.

In contrast, the biofilms exhibited a more complex thermal degradation profile, characterized by three distinct decomposition stages, indicative of greater structural complexity and enhanced interactions among the matrix components. All biofilm formulations showed an initial degradation temperature (Td_5_%) above 239 °C, reflecting a substantial improvement in thermal stability compared with the pure carrageenan sample. The control biofilm (without bioactive compounds) displayed three main decomposition peaks at 256 °C (Td_1_), 279 °C (Td_2_), and 448 °C (Td_3_), with the degradation process completing at approximately 513 °C and a total mass loss of 91%.

The incorporation of curcumin or synthetic curcuminoids did not markedly alter the overall thermal degradation profile but induced subtle shifts in the decomposition behavior. The CUR-loaded film exhibited slightly higher degradation temperatures (Td_5_% at 252 °C, Td_1_ at 277 °C, and Td_2_ at 373 °C), indicating enhanced thermal resistance, likely resulting from intermolecular interactions between the polymer matrix and curcumin molecules. Similarly, the CN56-loaded film displayed multiple decomposition events at 241 °C, 271 °C, 359 °C, and 448 °C, with the degradation process completing at approximately 520 °C and a total mass loss of 91%.

Although minor variations were observed in the decomposition temperatures, all formulations followed a comparable thermal degradation pathway, reinforcing that the structural framework of the films remained primarily governed by carrageenan as the main component. These findings suggest that incorporation of the inclusion complexes slightly improves the overall thermal stability of the system without altering its fundamental degradation mechanism.

These findings are consistent with previous reports indicating that the incorporation of cyclodextrin complexes enhances the thermal stability of polymeric films by promoting a more homogeneous dispersion of the active compounds and reducing volatile losses at lower temperatures [21]. Compared with PVA/carrageenan systems lacking inclusion complexes [5], the observed increase in thermal resistance reinforces the hypothesis that β-CD/curcuminoid complexes are effectively integrated within the polymeric network, thereby improving the overall robustness of the material during processing and storage.

Overall, the TGA results demonstrate that the incorporation of β-CD inclusion complexes does not compromise the films’ thermal integrity and may, in fact, enhance their stability under elevated temperatures. The improved thermal stability further supports the suitability of these biofilms for pharmaceutical applications, ensuring greater resilience throughout manufacturing, handling, and long-term storage.

### 2.4. Differential Scanning Calorimetry (DSC)

Differential scanning calorimetry (DSC) was conducted to evaluate the thermal behavior of the samples and identify potential phase transitions. The pure carrageenan sample exhibited no first-order thermal events—such as melting or crystallization—within the analyzed temperature range (−80 °C to 150 °C), confirming its predominantly amorphous nature under these conditions. The absence of discernible glass-transition or endothermic/exothermic peaks is consistent with previous reports for native carrageenan [28].

**Figure 2:**
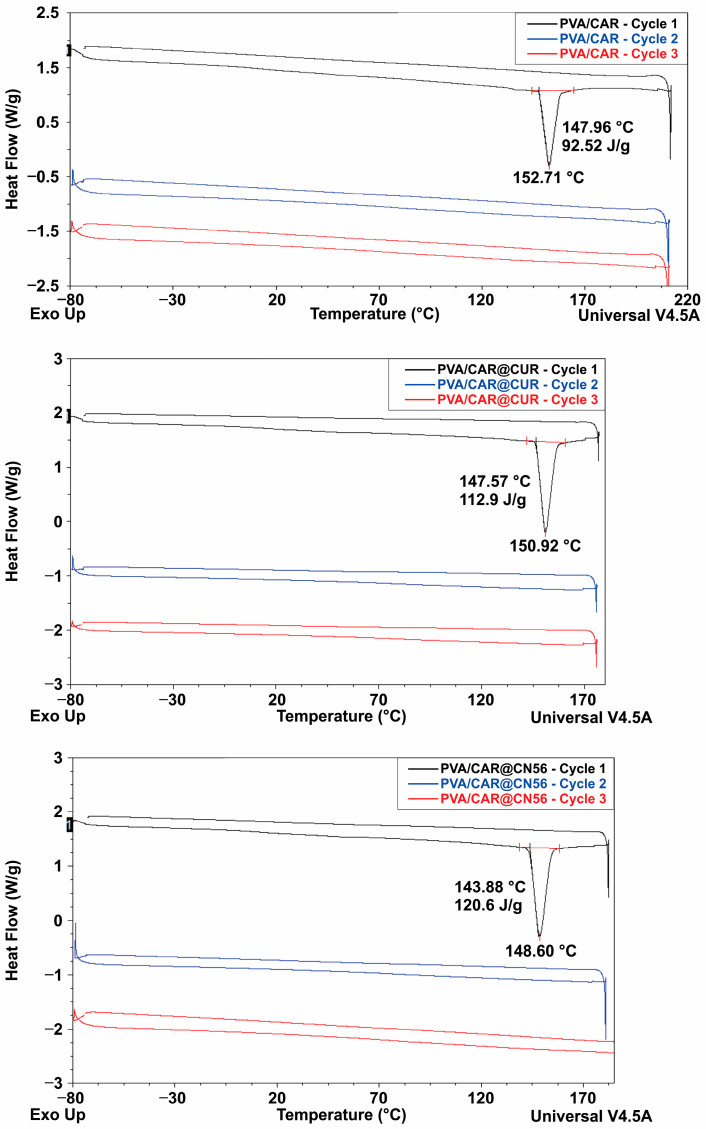
DSC thermogram of the PVA/CAR, PVA/CAR@CUR, and PVA/CAR@CN56 films.

In contrast, the DSC thermograms of the developed biofilms (PVA/CAR and PVA/CAR-based films loaded with CUR and CN56 inclusion complexes) displayed distinct thermal features. All three formulations exhibited exothermic melting transitions during the first heating cycle, with melting temperatures (Tm) observed at approximately 153 °C for the pure film, 151 °C for the CUR-loaded film, and 149 °C for the CN56-loaded film. These transitions were accompanied by enthalpy changes of 92.52 J g^−1^, 112.9 J g^−1^, and 120.6 J g^−1^, respectively (Figure 2).

The progressive decrease in melting temperatures and increase in enthalpy values suggest that incorporating curcuminoids promotes reorganization of the polymer net-work, potentially disrupting crystalline domains and facilitating thermal relaxation of polymer chains. This interpretation aligns with the observed reduction in crystallinity in FTIR and supports the hypothesis that inclusion complexes interfere with the ordered packing of PVA and carrageenan.

Importantly, no melting or other first-order thermal transitions were detected during the second heating cycle in any of the formulations, indicating irreversible amorphization following the initial thermal exposure. This behavior is consistent with previous findings for physically crosslinked PVA-based hydrogels, where thermal cycling induces permanent structural rearrangements [5]. Compared with other polymeric systems incorporating β-CD [21,23,29], the observed irreversible melting supports the notion that inclusion complexes modulate both the thermal and supramolecular characteristics of the polymer matrix, thereby enhancing its structural stability during processing and storage.

Furthermore, the irreversible amorphization observed may contribute to improved stability of the encapsulated bioactive compounds under biological conditions, favoring a more controlled and sustained drug release—an advantageous feature for localized therapeutic applications.

### 2.5. Morphological Analysis (SEM)

**Figure 3:**
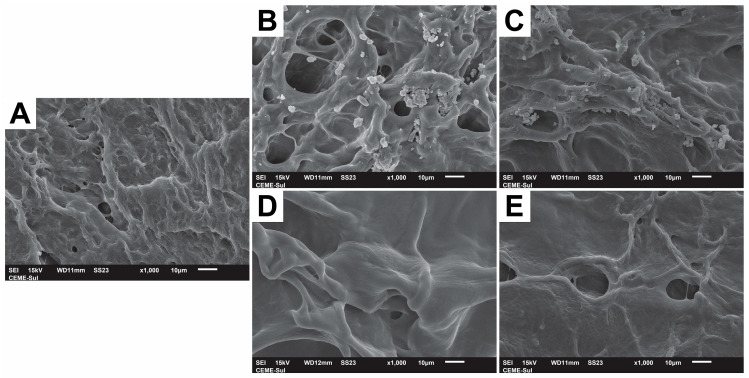
Scanning electron micrographs (1000× magnification) of the biofilm surfaces: (**A**) PVA/CAR; (**B**) PVA/CAR@CUR; (**C**) PVA/CAR@CN56; (**D**) PVA/CAR@β-CD/CUR; (**E**) PVA/CAR@β-CD/CN56.

The morphology of the prepared films was analyzed using scanning electron microscopy (SEM). The pure PVA/carrageenan film exhibited a homogeneous and slightly textured surface without evidence of phase separation, indicating high compatibility between the PVA and carrageenan components (Figure 3A). The compact morphology and absence of visible pores suggest that the freeze–thaw processing followed by drying promoted efficient polymer chain packing and the formation of a dense, uniform matrix.

Films loaded with free curcumin (PVA/CAR@CUR) and free CN56 (PVA/CAR@CN56) were also examined for comparison. The CUR-loaded film exhibited a more wrinkled and irregular surface (Figure 3B), whereas the CN56-loaded film presented a comparatively smoother morphology (Figure 3C). In both cases, discrete surface agglomerates were observed, likely corresponding to unincorporated curcuminoid crystals. These features suggest incomplete integration of the free active compounds within the polymeric network, possibly due to limited compatibility and dispersion during film formation.

In contrast, films containing β-CD inclusion complexes exhibited distinct morphological features. The PVA/CAR@β-CD/CUR film showed increased surface roughness and the presence of irregular microdomains (Figure 3D), likely arising from localized interactions between the β-CD/CUR complexes and the surrounding polymer matrix. Conversely, the PVA/CAR@β-CD/CN56 film presented a more uniform and smoother surface (Figure 3E), suggesting enhanced dispersion and compatibility of the β-CD/CN56 complexes within the hydrogel network. Similar trends have been reported in systems where cyclodextrin inclusion complexes mitigate aggregation and improve overall homogeneity in polymeric films [21].

These morphological differences are consistent with the FTIR and thermal analyses, supporting the hypothesis that the inclusion complexes disrupt polymer chain packing and facilitate more homogeneous incorporation of curcuminoids within the matrix. Compared with previous studies on PVA/carrageenan hydrogels lacking inclusion complexes [5], the improved surface uniformity observed here further reinforces the potential of this strategy to enhance the physicochemical performance of bioactive-loaded hydrogels. The homogeneous dispersion of bioactive compounds is particularly advantageous for achieving uniform release profiles and consistent therapeutic efficacy in vaginal applications.

### 2.6. Swelling Behavior

The swelling behavior of the films was evaluated in phosphate-buffered saline (PBS, pH 7.4), revealing pronounced differences among the formulations. The pure film, without incorporation of inclusion complexes, exhibited the lowest swelling capacity, reaching 473% after 120 min. In contrast, the films containing β-CD inclusion complexes demonstrated significantly higher swelling ratios. The PVA/CAR@β-CD/CUR film reached 881% after 75 min, whereas the PVA/CAR@β-CD/CN56 film displayed the highest swelling capacity, attaining 1080% within 45 min, of which 1028% occurred during the first 10 min.

The substantial increase in fluid uptake observed for the loaded films can be attributed to the disruptive effect of the inclusion complexes on the polymeric network [30]. These complexes act as physical spacers, disturbing the dense arrangement of PVA and carrageenan chains established during freeze–thaw crosslinking and subsequent drying. As a result, the presence of the inclusion complexes increases the free volume within the matrix, enhances porosity, and facilitates water diffusion throughout the hydrogel structure. Moreover, the intrinsic hydrophilicity of PVA and carrageenan further promotes fluid absorption, while the reduced crystallinity detected by FTIR analysis supports the observed network expansion and improved polymer chain mobility [5,24].

Structural differences and specific molecular interactions between the β-CD/CUR and β-CD/CN56 complexes may account for the distinct swelling behaviors observed. The β-CD/CN56 complex appears to induce a more pronounced disruption of the polymeric matrix, possibly due to enhanced compatibility or weaker intermolecular interactions, leading to a less compact network structure. This elevated swelling capacity, particularly in the CN56-loaded films, is advantageous for local drug delivery systems (LDDSs), as it facilitates rapid matrix hydration and accelerates the release of the active compound—provided that mechanical integrity is maintained.

In summary, the incorporation of β-CD inclusion complexes markedly enhances the swelling properties of the films, and fine-tuning the matrix composition represents a promising approach for modulating drug release behavior in hydrogel-based delivery systems [22].

The differences observed between the CUR- and CN56-based systems may be attributed to specific molecular interactions and varying degrees of compatibility of their respective inclusion complexes with the polymeric matrix. The β-CD/CN56 complex appears to induce a more pronounced disruption of the polymer network, leading to faster and greater swelling. Similar behavior was reported by Croitoru et al. [5], who observed enhanced swelling when hydrophilic additives disrupted the packing of PVA/carrageenan chains, although the absolute swelling values in the present study were substantially higher. These findings suggest that inclusion complexation represents an effective strategy for modulating hydrogel swelling behavior beyond what is typically achieved using uncomplexed additives [27].

This property is particularly relevant in the context of topical delivery systems, where rapid swelling facilitates intimate contact between the hydrogel and the application site, thereby enhancing bioadhesion, local hydration, and diffusion of the drug into the target tissue. In addition, high swelling capacity can improve patient comfort by maintaining a moist microenvironment conducive to wound healing and by reducing transepidermal water loss [31]. Previous studies have demonstrated that physically crosslinked hydrogels with greater swelling ratios often exhibit more sustained release profiles, owing to the formation of interconnected pores that act as diffusion pathways for the gradual release of bioactive compounds [5,21].

Therefore, the assessment of swelling behavior provides critical insights into both the structural performance of the hydrogel and its suitability for prolonged topical applications. Although the observed swelling and hydration behavior suggest favorable interaction with mucosal tissues, no mucoadhesion assays were conducted in this study. Accordingly, these findings should be interpreted as indicative of potential—rather than confirmed—mucoadhesive performance.

### 2.7. Release Profile and Kinetics

To evaluate the potential of the PVA/CAR@β-CD/CUR and PVA/CAR@β-CD/CN56 films as controlled drug delivery systems, the release profiles of the incorporated compounds were assessed in phosphate-buffered saline (PBS, pH 7.4) containing ethanol at 37 °C [19]. This hydroalcoholic medium was selected to facilitate the dissociation of CUR and CN56 from their β-cyclodextrin (β-CD) inclusion complexes. As previously discussed, the hydrophobic cavity of β-CD provides a favorable microenvironment for stabilizing CUR molecules, while its hydrophilic exterior enhances their apparent solubility in aqueous media. However, inclusion complexation modifies the UV–Vis absorbance spectrum of CUR, thereby hindering its direct quantification in PBS alone. To address this limitation, a small volume of ethanol was added to the PBS solution, serving as both a solubilizing and stabilizing agent and enabling accurate spectrophotometric detection of CUR and CN56 [29,31,32,33].

**Figure 4:**
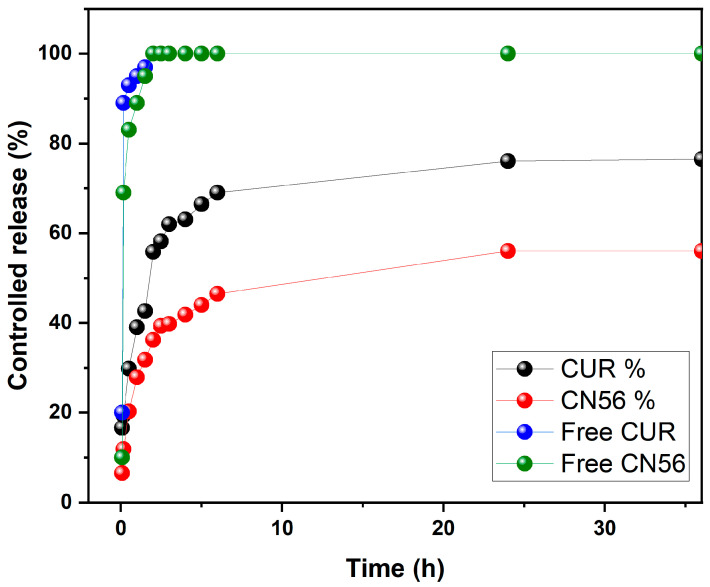
Cumulative release profiles of encapsulated and free CUR and CN56 over time in PBS pH 7.4 at 37 °C.

A sudden release of bioactive compounds can result in high initial doses, potentially causing local or systemic toxicity due to nonspecific action. In contrast, the films developed in this study exhibited controlled and sustained release profiles, thereby minimizing such risks. As shown in Figure 4, after 1.5 h of incubation, only 42.61% of encapsulated CUR and 31.79% of CN56 had been released, whereas their free counterparts (not incorporated into films) achieved complete release within the same time frame. This pronounced difference underscores the ability of the films to prevent burst release and promote gradual diffusion of the encapsulated compounds.

In both formulations, the release progressed steadily and reached equilibrium after approximately 6 h, with final cumulative release values of 76.49% for CUR and 56.02% for CN56. These results confirm the films’ capacity to provide controlled and prolonged drug delivery, while the complexation with β-CD contributes to enhanced compound stability and solubility under physiological conditions—attributes particularly advantageous for localized drug delivery systems (LDDS).

For the CN56 system, the first-order kinetic model yielded R^2^ = 0.922 (Q_e_q = 52.57; K_1_ = 0.58), while the Korsmeyer–Peppas model produced R^2^ = 0.919 with a diffusional exponent of *n* = 0.257, indicating a predominant Fickian diffusion mechanism. This observation is consistent with the swelling behavior of the PVA/CAR@β-CD/CN56 film, which exhibited a rapid 1080% fluid uptake within 45 min, generating a steep concentration gradient that promoted diffusion even at early stages of the release process.

The high loading efficiencies achieved (75.62% for CUR and 79.00% for CN56), together with the distinct swelling behaviors and controlled release kinetics, underscore how the incorporation of β-CD inclusion complexes modulates the release dynamics. In the CUR-loaded system, moderate swelling facilitated gradual diffusion, whereas in the CN56-loaded system, rapid swelling promoted faster diffusion through a more porous matrix. Similar correlations between swelling capacity and Fickian-controlled release have been reported in other β-CD-based hydrogel systems [33].

These findings corroborate the structural and swelling data presented in earlier sections, demonstrating that the inclusion complexes modulate polymer chain packing, enhance porosity, and reduce crystallinity. Collectively, the thermal, morphological, and kinetic analyses consistently support the hypothesis that β-CD/curcuminoid complexes induce supramolecular reorganization within the hydrogel matrix, facilitating efficient drug loading and diffusion-controlled release. Such properties are particularly advantageous for topical drug delivery platforms, where prolonged residence time, effective local retention, and suppression of burst release are essential for therapeutic efficacy and patient compliance. The sustained, diffusion-controlled release observed in this study aligns well with the therapeutic requirements for the treatment of bacterial vaginosis, in which extended local drug activity is crucial for achieving optimal outcomes.

### 2.8. Rheological Behavior and Viscosity Profile

**Figure 5:**
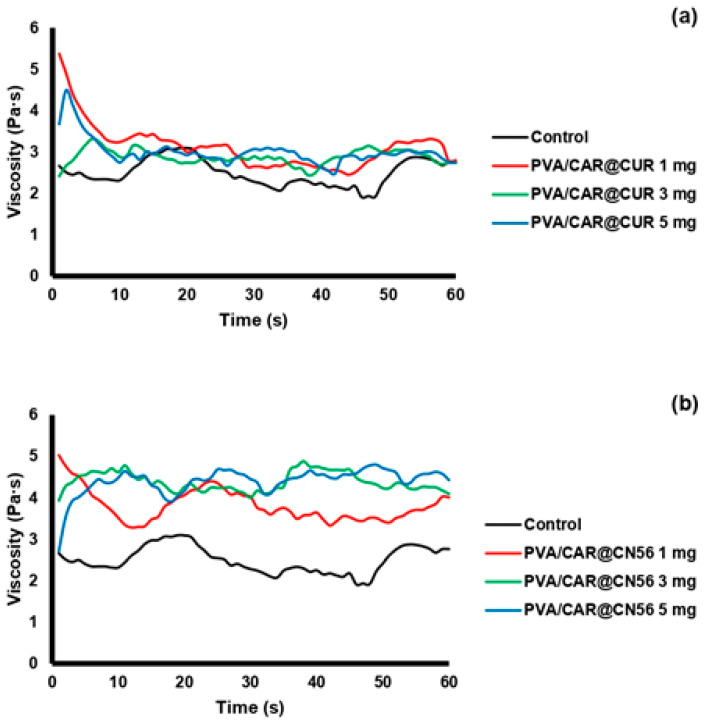
Rheological profile of (**a**) PVA/CAR@CUR and (**b**) PVA/CAR@CUR at 1, 3, and 5 mg concentrations compared with the control sample.

The rheological analysis demonstrated that the incorporation of curcumin (CUR) and the synthetic curcuminoid CN56 distinctly affected the viscosity and structural organization of the carrageenan/PVA hydrogels (Figure 5). Under constant shear (10 s^−1^), the CUR-loaded samples exhibited a transient viscosity peak followed by rapid decay and stabilization around 3.0 Pa·s, which was only slightly higher than the control (≈2.5 Pa·s). This mild and non-linear thickening behavior suggests weak interactions between CUR and the polymeric network, likely due to the poor aqueous solubility and partial aggregation of curcumin within the matrix. Similar rheological trends have been reported for cyclodextrin–curcumin hydrogels, where heterogeneous distribution of CUR led to limited polymer–drug entanglement and reduced network homogeneity [34,35].

In contrast, CN56-incorporated hydrogels exhibited a sustained and markedly higher viscosity (≈3.8–4.3 Pa·s), maintaining stability throughout the measurement period. This consistent thickening effect is indicative of enhanced internal structuring and stronger intermolecular interactions with the carrageenan/PVA chains. The higher polarity and molecular rigidity of CN56 may facilitate hydrogen bonding and electrostatic association with sulfate groups from carrageenan and hydroxyl groups from PVA, resulting in more cohesive polymeric domains. Comparable reinforcement behavior has been described in PVA-based hydrogels containing phenolic or β-cyclodextrin complexes, in which increased polarity of the guest molecule promoted supramolecular organization and higher viscoelastic resistance [34,35].

**Figure 6:**
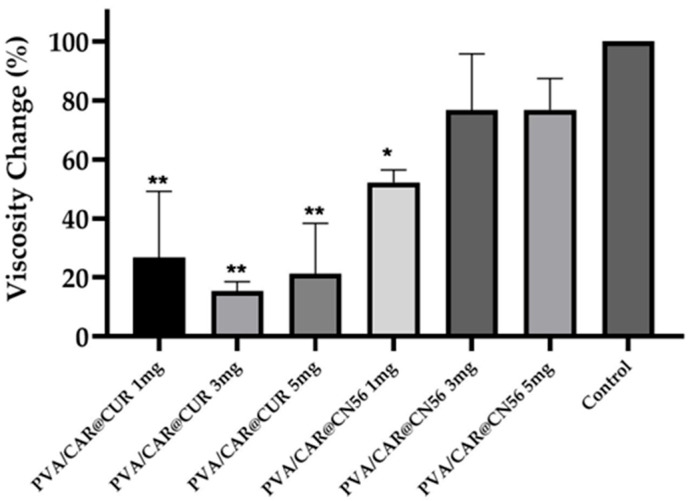
Mean percentage change in viscosity relative to the control, calculated as the average of the different concentrations (1, 3, and 5 mg) for PVA/CAR@CUR and PVA/CAR@CN56. Asterisks indicate statistical significance compared to the control group (*p* < 0.05 for * and *p* < 0.01 for **).

Quantitatively, CN56-loaded formulations presented an average viscosity enhancement of approximately 45% relative to the control, whereas CUR systems showed only a modest 15% increase (Figure 6). The PVA/CAR@CN56 formulations also exhibited a concentration-dependent rise in viscosity, reaching values close to the control level at 3–5 mg, while CUR-loaded samples showed only minor variations. Statistical analysis confirmed that CN56 produced a significantly higher thickening effect (*p* < 0.05 or *p* < 0.01) than CUR at equivalent concentrations.

These results corroborate previous findings by Cerveira et al. [36], who demonstrated that synthetic monocurcuminoids such as CN63 and CN67 exhibit superior chemical stability and stronger molecular interactions compared with native curcumin, due to the absence of the central β-diketone moiety responsible for tautomerization and self-aggregation [36]. The improved rheological response of CN56 thus likely arises from reduced hydrophobic clustering and enhanced compatibility with the PVA/carrageenan matrix.

From a functional standpoint, the observed viscosity increase implies greater structural organization and resistance to flow, which are desirable for biomedical and vaginal delivery systems. Enhanced viscosity is closely associated with improved mucoadhesion and prolonged residence time at the site of application, as reported for PVA- or Pluronic-based hydrogels containing cyclodextrin–polyphenol complexes [34,35]. Therefore, the CN56-reinforced hydrogel may offer superior mechanical stability and sustained release behavior compared with the native curcumin formulation.

Overall, these findings indicate that structural modification of curcumin into the CN56 analogue not only enhances its dispersion and molecular compatibility within the carrageenan/PVA network but also confers improved rheological stability, potentially translating into greater mucoadhesive performance and bioactive retention in topical vaginal applications.

### 2.9. Biological Evaluation

#### 2.9.1. Minimum Inhibitory and Bactericidal Concentrations (MIC and MBC)

The antimicrobial activity of curcumin (CUR) and the synthetic curcuminoid CN56 was evaluated against *Gardnerella vaginalis* (ATCC 14018) and a clinical isolate of *Candida albicans*. The results are summarized in Table 2.

Both compounds exhibited inhibitory and bactericidal activity under the tested conditions, with curcumin showing markedly higher potency than CN56 against both micro-organisms. For *G. vaginalis*, CUR inhibited bacterial growth at a concentration of 0.3125 µg µL^−1^, whereas CN56 required 10 µg µL^−1^ to achieve the same effect. A similar pattern was observed for *C. albicans*, where CUR demonstrated MIC and MBC values of 0.625 µg µL^−1^ compared with 5 µg µL^−1^ for CN56. The identical MIC and MBC values obtained for each compound indicate a bactericidal effect, in which the concentrations required to inhibit visible growth were also sufficient to eliminate viable cells.

These results reinforce the well-established antimicrobial potential of curcumin, attributed to its capacity to disrupt microbial membranes, interfere with enzymatic activity, and inhibit quorum sensing and biofilm formation [23,29,37]. Although CN56 exhibited lower activity than CUR, it still demonstrated inhibitory effects at relatively low concentrations, confirming that chemical modification of curcuminoids can preserve antimicrobial capacity while potentially improving physicochemical stability.

Overall, the MIC and MBC data confirm that both CUR and CN56 possess intrinsic antimicrobial properties suitable for incorporation into carrageenan/PVA hydrogels, enabling subsequent evaluation of the formulations’ ability to deliver these compounds in a controlled and sustained manner.

#### 2.9.2. Antimicrobial Activity: Time-Kill Assay

The time-kill assays were conducted to evaluate the antimicrobial potential of carrageenan-based biofilms and their composites with curcumin and curcuminoids against *Gardnerella vaginalis*. Two strains were tested: the ATCC 14018 reference strain and a clinical isolate (CI).

**Figure 7:**
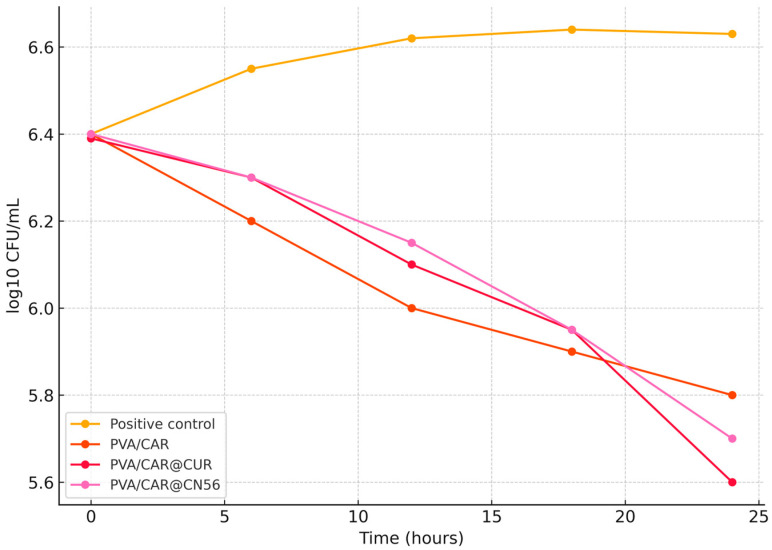
Time-kill curve of *Gardnerella vaginalis* ATCC 14018 treated with PVA/CAR, PVA/CAR@CUR, and PVA/CAR@CN56 over 24 h.

**Figure 8:**
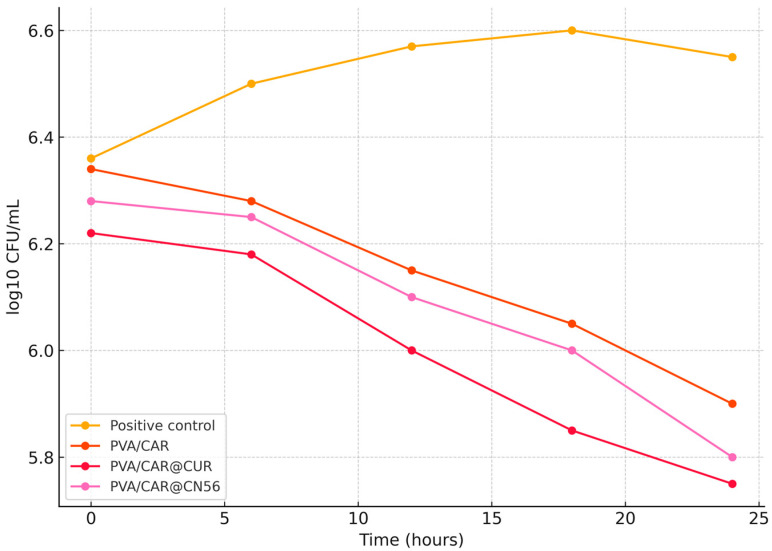
Time-kill curve of *Gardnerella vaginalis* clinical isolate treated with PVA/CAR, PVA/CAR@CUR, and PVA/CAR@CN56 over 24 h.

For both strains, the untreated group (positive control) exhibited a progressive increase in bacterial load over the 24 h period, confirming cell viability and normal growth under the experimental conditions (Figure 7 and Figure 8). In contrast, the negative control (medium without inoculum) showed no detectable bacterial growth and was omitted from the figure to preserve scale and clarity. Treatment with biofilms incorporating CUR or CN56 resulted in a gradual decline in viable bacterial counts, particularly between 6 and 24 h, with final reductions ranging from approximately 0.5 to 0.8 log units relative to the initial time point.

Comparable effects have been reported by Cerveira et al. [36], who observed that synthetic monocurcuminoids (CN63, CN67, CN77) exhibited stronger antimicrobial activity than curcumin, attributed to greater molecular stability and enhanced interaction with bacterial membranes. Similarly, Sravani et al. [38] demonstrated that curcumin-sulfobutyl-ether-β-cyclodextrin complexes promoted sustained bacteriostatic effects, maintaining inhibition over time due to improved solubility and gradual release of the active compound. In agreement, Fernández-Romero et al. [35] also reported that curcumin encapsulated in polymeric β-cyclodextrin hydrogels preserved antimicrobial activity over extended incubation, reinforcing the role of inclusion complexation in stabilizing curcuminoids and facilitating controlled diffusion through the matrix. Together, these reports support the present findings, suggesting that inclusion complexation and matrix embedding are key factors for achieving prolonged and stable antimicrobial activity.

Interestingly, biofilms composed solely of carrageenan exhibited microbial contamination and low antimicrobial activity, reinforcing the essential role of the incorporated active agents. The superior effect observed for curcuminoids compared with curcumin may be attributed to their greater chemical stability and the broader spectrum of derivatives present in the curcuminoid extract, which likely facilitate prolonged interactions with bacterial membranes.

The observed reduction in viable cell counts over time indicates that the developed hydrogel films exhibit bacteriostatic to bactericidal behavior, depending on the formulation, exposure duration, and strain tested. These findings support the potential application of these materials for the prevention or adjunctive treatment of infections caused by *Gardnerella vaginalis*, a key pathogen associated with bacterial vaginosis.

#### 2.9.3. Hemolytic Activity

**Figure 9:**
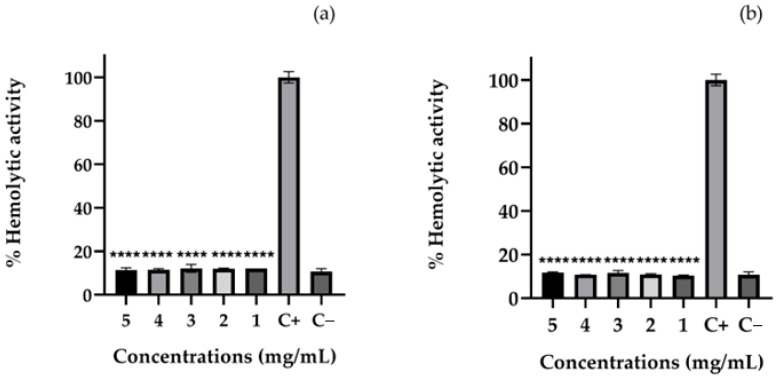
Hemolytic activity (%) of (**a**) PVA/CAR@CUR and (**b**) PVA/CAR@CN56 at different concentrations (1–5 mg/mL); positive control (C^+^: DMSO) and negative control (C^−^: PBS). Results are expressed as mean ± SD (*n* = 3). Asterisks indicate statistical significance compared to the negative control (PBS): *p* < 0.001 (****).

The hemolytic assay was performed to assess the potential cytotoxic effects of the PVA/carrageenan-based hydrogels on erythrocyte membranes. As shown in Figure 9, both PVA/CAR@CUR and PVA/CAR@CN56 exhibited very low hemolytic activity (10.4–12.2%) across all concentrations tested (1–5 mg mL^−1^), values statistically comparable to the negative control (PBS, 10.7 ± 1.4%). In contrast, the positive control (DMSO) induced complete hemolysis (100 ± 2.6%), confirming the sensitivity of the assay.

According to ISO 10993-5:2009 [39] and the classification proposed by Sæbø et al. [40], materials inducing <5% hemolysis are considered non-hemolytic and those between 5 and 25% are slightly hemolytic; thus, both formulations can be categorized as slightly hemolytic but hemocompatible.

The obtained results align well with prior studies demonstrating the good hemocompatibility of curcumin- and cyclodextrin-based hydrogels. For instance, Wangsawangrung et al. [34] reported negligible hemolysis (<10%) for PVA/HP-β-CD hydrogels loaded with quercetin, attributing the mild response to polymer shielding and the antioxidant nature of the incorporated polyphenol. Similarly, Fernández-Romero et al. [35] found that curcumin complexed with polymeric β-cyclodextrin (EpiβCD) and embedded in Pluronic/hyaluronate gels exhibited no significant hemolytic or irritant effects, confirming the protective role of the cyclodextrin matrix in mitigating membrane damage.

In agreement with these findings, Sravani et al. [38] also demonstrated that a curcumin–sulfobutyl-ether-β-cyclodextrin inclusion complex caused <15% hemolysis in vitro, reinforcing the biocompatibility of cyclodextrin-based curcuminoid systems. The low hemolytic percentages observed in the present study therefore suggest that both CUR and CN56 are well-tolerated by erythrocytes and that their incorporation within the PVA/carrageenan matrix does not promote lysis or membrane destabilization.

Interestingly, these results parallel those obtained by Cerveira et al. [36], who evaluated the hemolytic activity of several synthetic monocurcuminoids (CN63, CN67, CN77) and reported non-cytotoxic behavior with hemolysis values below 15%. This consistency reinforces the notion that structural modification of curcumin, such as removal of the β-diketone bridge, improves molecular stability and reduces non-specific interactions with biological membranes. Accordingly, CN56, which follows the same monocurcuminoid scaffold, maintained similar biocompatibility while exhibiting improved physicochemical properties within the hydrogel network.

Taken together, these observations indicate that both CN56 and CUR hydrogels are hemocompatible, exhibiting only slight baseline hemolysis likely due to optical interference or minor sample residues rather than true membrane rupture. Their overall safety profile supports the potential of these formulations for mucosal or intravaginal applications, where direct contact with epithelial and vascular tissues is expected.

#### 2.9.4. Cell Viability Assay (MTT Reduction)

**Figure 10:**
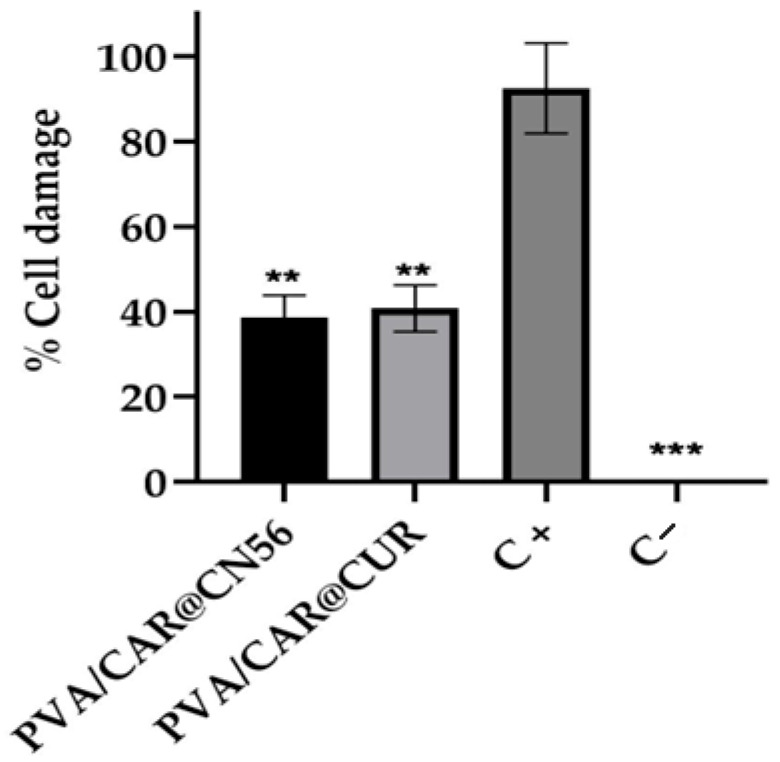
Cell viability (%) of HeLa cells exposed to films containing curcumin (CUR) and curcuminoid (CN56) (5 mg/mL) for 24 h; positive control (C^+^: DMSO) and negative control (C^−^: untreated cells). Data are expressed as mean ± SD (*n* = 3). Asterisks indicate statistical significance compared to the positive control (C^+^: DMSO): *p* < 0.01 (**), and *p* < 0.001 (***).

The cytotoxicity of curcumin (CUR) and curcuminoid (CN56) films was evaluated in HeLa cells using the MTT reduction assay after 24 h of exposure (Figure 10). Both formulations exhibited cell viabilities comparable to the negative control, confirming the absence of overt cytotoxic effects under the tested conditions. Mean cell viability values for CUR and CN56 were 48.6 ± 10.1% and 39.3 ± 9.6%, respectively, while untreated control cells presented 51.5 ± 6.3%. The positive control (DMSO) induced marked cell damage (≈93%), validating the assay performance.

Although the viability values were slightly below the 70% threshold defined by ISO 10993-5:2009 [39] for non-cytotoxic materials, these moderate reductions are consistent with the metabolic behavior commonly observed in polyphenol-containing biomaterials. Curcumin and its analogs are known to modulate mitochondrial dehydrogenase activity and redox balance, which can transiently decrease MTT conversion without causing actual membrane or DNA damage. Thus, the reduction in formazan formation here likely reflects antioxidant or redox interference rather than true cytotoxicity.

Comparable effects were reported by Wangsawangrung et al. [34], who observed slight decreases in MTT signal for PVA/HP-β-CD hydrogels loaded with quercetin, attributed to reversible redox modulation. Similarly, Fernández-Romero et al. [35] demonstrated that curcumin encapsulated in Epi-β-cyclodextrin hydrogels maintained cell viability above 60%, confirming the non-cytotoxic profile of curcumin inclusion complexes. In another study, Sravani et al. [38] reported that curcumin–sulfobutyl-ether-β-cyclodextrin complexes retained >80% viability in fibroblasts, further emphasizing that cyclodextrin complexation and polymer embedding improve curcumin biocompatibility.

The present results are also strongly corroborated by Cerveira et al. [36], who tested structurally related synthetic monocurcuminoids (CN63, CN67, CN77) and reported half-maximal inhibitory concentration (IC_50_) values between 49 and 130 µM, indicating low cytotoxicity in mammalian cells. The similar non-toxic profile of CN56 observed here reinforces the conclusion that monocarbonyl curcuminoids maintain biocompatibility while exhibiting enhanced physicochemical stability.

Taken together, these findings demonstrate that both CUR and CN56 hydrogels exhibit satisfactory biocompatibility and minimal cytotoxic response, in agreement with the hemolytic results discussed previously. The minor decrease in metabolic activity likely stems from reversible redox interactions rather than cellular damage, supporting the safety of these formulations for mucosal and localized drug delivery applications.

## 3. Conclusions

This study reports the development and characterization of carrageenan/PVA hydrogel films incorporating curcumin and a synthetic curcuminoid (CN56) through β-cyclodextrin inclusion complexes. Physicochemical and thermal analyses indicated effective incorporation of the bioactive compounds, while morphological evaluation revealed uniform distribution and compatibility within the polymeric matrix. Swelling and release assays suggested a favorable profile for sustained and controlled delivery of curcuminoids.

The antimicrobial assays demonstrated activity of the developed films against *Gardnerella vaginalis* and *Candida albicans*, supporting the potential of these formulations for topical applications in women’s health. Additionally, complementary biocompatibility evaluations, including hemolytic activity and MTT reduction assays, indicated low cytotoxicity and satisfactory hemocompatibility, reinforcing the safety of the developed systems. Rheological analysis further suggested enhanced viscosity and potential mucoadhesive properties, which are advantageous for local retention and prolonged release.

Although the results suggest an improvement in antimicrobial performance upon bioactive incorporation, further comparative studies—including free curcumin and curcuminoids, as well as long-term stability and in vivo assessments—are required to confirm these effects. Overall, the findings highlight the feasibility of carrageenan-based hydrogels as multifunctional, biocompatible biomaterials with promising applicability in vaginal drug delivery systems.

## 4. Materials and Methods

### 4.1. Materials

Poly(vinyl alcohol) (PVA, 98% hydrolyzed, Mw 124,000 g/mol), sodium hydroxide (NaOH), β-cyclodextrin (β-CD), and curcumin (CUR) from *Curcuma longa* were purchased from Sigma-Aldrich (St. Louis, MO, USA). Ethanol (P.A.) and hydrochloric acid (HCl) were obtained from Synth (São Paulo, Brazil). All reagents were of analytical grade and used without further purification. The synthetic curcuminoid CN56 was kindly provided by the Bioforensics Research Group (Federal University of Pelotas, Pelotas, Brazil) and synthesized according to the Claisen–Schmidt condensation method previously described by Silva et al. [41].

### 4.2. Macroalgae Sampling

Red macroalgae *Gigartina skottsbergii* were manually collected from the eulittoral or infralittoral zones at various locations along the Antarctic Peninsula between November and December 2015 during the Thirty-Fourth Brazilian Antarctic Expedition. Samples were rinsed with seawater and then with distilled water to remove impurities, microorganisms, and salts. After morphological identification, the specimens were lyophilized.

### 4.3. Carrageenan Extraction

Carrageenan was extracted following the method described by Webber et al. [42], with modifications Zank et al. [3]. Ten grams of dried *G. skottsbergii* were rehydrated in 800 mL of distilled water for 1 h and extracted in a water bath at 60 °C for 4 h. The solution was vacuum-filtered, and the carrageenan was obtained by drying the filtrate at 40 °C for 72 h.

### 4.4. Preparation of Inclusion Complexes

Inclusion complexes of β-CD with CUR or CN56 were prepared based on the procedure described by Gerola et al. [21], with adaptations. First, an aqueous solution of β-CD was prepared, and its pH was adjusted to 5. A stock solution of CUR or CN56 (5 mg/mL in acetone) was then added dropwise to the β-CD solution to achieve a final molar ratio of 1:6 (curcuminoid:β-CD). To ensure initial homogeneity, the resulting mixture was immediately sonicated in an ultrasonic bath for 5 min. Subsequently, the suspension was maintained under continuous magnetic stirring (200 rpm) at room temperature (25 ± 2 °C) for 48 h to facilitate the complexation process. The formation of the inclusion complex was monitored by collecting aliquots of the supernatant at specific time intervals and analyzing them with a UV-Vis spectrometer (IL–592, Kasuaki, Uberlândia, Brazil). The loading efficiency (LE) was determined by quantifying the amount of uncomplexed curcuminoid remaining in the supernatant using the following Equation (1):

LE (%) = (([Cur]_initial_ − [Cur]_t_)/[Cur]_initial_) × 100 (1)

### 4.5. Hydrogel Films Preparation

The hydrogel films were fabricated using a physical crosslinking method based on freeze–thaw cycles. To prepare hydrogel films, 100 mg of carrageenan was dissolved in 5 mL of distilled water at 60 °C under constant magnetic stirring. Separately, a PVA solution was prepared by dissolving 1000 mg of PVA in 15 mL of distilled water at 80 °C for 3 h, also under magnetic stirring, until a clear and homogeneous solution was obtained. Subsequently, the two polymer solutions were blended and stirred for 30 min at room temperature to form a homogeneous precursor solution. Following the procedure adapted from Gularte et al. [24], the pH of this blend was adjusted to 5. The previously prepared aqueous suspension containing the β-CD/CUR or β-CD/CN56 inclusion complex (from Section 4.4) was then added to the polymer blend. The final mixture was gently stirred at 100 rpm for an additional 30 min to ensure a uniform dispersion of the complexes. The homogeneous solution were poured into Petri dishes (85 × 10 mm).

To induce physical crosslinking, the dishes were first subjected to a pre-freezing step at −20 °C for 12 h. This was followed by five consecutive freeze–thaw cycles, each consisting of 1 h of freezing at −20 °C and 30 min of thawing at room temperature. After freeze–thawing, the films were lyophilized.

### 4.6. Swelling Test

Dried film samples were weighed and immersed in PBS (pH 7.4). The system was kept at 37 °C under constant agitation (100 rpm) in an orbital shaker. At pre-determined time intervals, the films were removed, excess surface liquid was blotted with absorbent paper, and they were weighed again. The swelling degree was calculated using Equation (2):

Swelling (%) = (W_s_ − W_d_)/W_d_ × 100 (2)

where W_s_ is the swollen weight and W_d_ is the initial dry weight. Measurements were performed in triplicate.

### 4.7. In Vitro Release of CUR and CN56

Film samples containing approximately 90 µg of CUR or CN56 were immersed in an ethanol:PBS (10:90 *v*/*v*, pH 7.4) release medium at 37 °C under gentle agitation [43]. At predetermined intervals, a 3 mL aliquot was withdrawn, and an equal volume of fresh medium was added back to maintain sink conditions. The concentration of the released compound was determined by measuring the absorbance at 425 nm and calculated from a calibration curve (R^2^ ≈ 0.998). Kinetic modeling was performed using first-order and Korsmeyer–Peppas Equations (3) and (4) [44,45]:

Q_t_ = Q_eq_ (1 − e^−k^_1_^t^) (First-order model) (3)

Q_t_ = K_k_ t^n^ (Korsmeyer-Peppas model) (4)

where Q_t_ is the amount released at time t, Q_eq_ is the maximum release, K_1_ is the first-order constant, and *n* is the release exponent.

### 4.8. FTIR Spectroscopy

Fourier-transform infrared (FTIR) spectra were acquired using spectrometer (Shimadzu Affinity, Kyoto, Japan) over the 400–4000 cm^−1^ range, with 64 scans per sample using the KBr pellet method.

### 4.9. Scanning Electron Microscopy (SEM)

Surface morphology was assessed by scanning electron microscopy (SEM) using a JEOL JSM-6610LC microscope (Tokyo, Japan) at 15 kV. Lyophilized film samples were sputter-coated with gold before imaging.

### 4.10. Thermogravimetric Analysis (TGA)

Thermogravimetric analysis (TGA) was performed using a TGA Q5000 thermobalance (TA Instruments Inc., New Castle, DE, USA). Calibration was carried out using calcium oxalate monohydrate (CaC_2_O_4_·H_2_O, 99.9%). Approximately 5 mg of the sample was placed in a platinum pan and heated from room temperature to 600 °C at a constant heating rate of 10 °C·min^−1^ under an inert nitrogen atmosphere (50 mL·min^−1^). No prior drying step or isothermal hold was applied before the analysis. Data acquisition and analysis were conducted using TA Universal Analysis 2000 software, version 4.5 (TA Instruments Inc., New Castle, DE, USA).

### 4.11. Differential Scanning Calorimetry (DSC)

Thermal transitions were determined by Modulated Temperature Differential Scanning Calorimetry (MTDSC) using a DSC Q2000 instrument (TA Instruments Inc., New Castle, DE, USA). Approximately 3 mg of the sample was accurately weighed using a Sartorius M500P analytical balance (Sartorius AG, Göttingen, Germany) (±0.001 mg). Analyses were performed under a nitrogen flow of 50 mL·min^−1^. The samples were subjected to two heating and cooling cycles between −80 °C and 150 °C, at a heating/cooling rate of 5 °C·min^−1^. The modulation amplitude was set to ±1 °C with a modulation period of 60 s. The first heating/cooling cycle was used to eliminate thermal history, and the second was used to determine the glass transition and melting-related events. Data acquisition and analysis were carried out using TA Universal Analysis 2000 software, version 4.5 (TA Instruments Inc., New Castle, DE, USA).

### 4.12. Rheological Analysis

The rheological behavior of the hydrogel formulations was evaluated using a Brookfield RS-CPS+ Rheometer (Brookfield Engineering Laboratories, Inc., Middleboro, MA, USA) equipped with a C 50-1 spindle and controlled by Rheo 3000 software (version 1.2). Samples were analyzed at room temperature (25 ± 1 °C) to determine viscosity and flow characteristics. Measurements were conducted at a constant shear rate of 10 s^−1^ for 60 s. The viscosity values were recorded in millipascal-seconds (mPa·s) and expressed as the mean of triplicate measurements.

The method followed a standardized protocol for viscosity assessment of xanthan gum-based systems, adapted to hydrogel samples [46]. Briefly, hydrogel aliquots were placed directly on the measurement plate, ensuring full contact between the spindle and the sample. The rheological stability of the system was confirmed by comparing viscosity values over time, verifying the absence of structural degradation or phase separation during the measurement period.

### 4.13. Antimicrobial Time-Kill Assay

*Gardnerella vaginalis* (ATCC 14018) and a clinical isolate (CI) were used, obtained from the LAPEBBIOM culture collection at the Federal University of Pelotas. Cultivation was performed on Columbia agar under anaerobic conditions at 37 °C for 24 h. Bacterial inocula were adjusted to 0.5 McFarland (1.5 × 10^8^ CFU/mL) in 0.9% NaCl solution.

Time-kill assays followed an adapted protocol from Appiah et al. [47]. In sterile test tubes, 1 mL of Mueller-Hinton broth and 1 g of each hydrogel formulation (CAR, CAR + CUR, CAR + CN56) were combined. Then, 100 μL of the standardized bacterial inoculum was added. Negative controls contained only broth; positive controls included broth and bacteria. Tubes were incubated at 37 °C with shaking, and aliquots were collected at 0, 6, 12, 18, and 24 h. Aliquots were plated on MH agar and incubated for 24 h at 37 °C. Bacterial growth was expressed as log CFU/mL over time.

### 4.14. Minimum Inhibitory Concentration (MIC) and Minimum Bactericidal Concentration (MBC)

The minimum inhibitory concentration (MIC) of curcumin and synthetic curcuminoids was determined by the broth microdilution method, following the Clinical and Laboratory Standards Institute (CLSI) guidelines (M7-A6) [48]. Assays were carried out in 96-well microplates, each well containing 100 μL of Mueller–Hinton (MH) broth (Kasvi, Paraná, Brazil). Serial dilutions of the compounds were prepared to a final volume of 100 μL per well. Subsequently, 10 μL of standardized bacterial suspension was added to each well. The bacterial growth control consisted of MH broth with inoculum, while the sterility control contained only MH broth. The plates were incubated at 37 °C for 24 h under aerobic conditions.

MIC was defined as the lowest concentration at which no visible bacterial growth (turbidity) was observed. To determine whether this concentration was bacteriostatic or bactericidal, samples from the wells corresponding to 0.5 × MIC, 1 × MIC, and 2 × MIC were plated on MH agar and incubated at 37 °C for 48 h. The minimum bactericidal concentration (MBC) was defined as the lowest concentration at which no bacterial colonies were detected under the tested conditions.

### 4.15. Hemolytic Activity Assay

The hemolytic activity assay was performed using defibrinated sheep blood (Laborclin, Pinhais, Brazil). The blood was centrifuged at 1500 rpm for 10 min, and the resulting plasma was discarded. The erythrocytes were resuspended in phosphate-buffered saline (PBS, pH 7.4) to a final concentration of 4%. Films containing curcumin (CUR) and curcuminoid (CN56) were tested at concentrations of 1, 2, 3, 4, and 5 mg/mL. Dimethyl sulfoxide (DMSO) and PBS were used as positive and negative controls, respectively. Aliquots (100 µL) of each solution were incubated in microtubes at 37 °C with gentle agitation (30 rpm) for 1 h. After incubation, samples were centrifuged at 800× *g* for 10 min, and 100 µL of the supernatant was transferred to a 96-well microplate. Absorbance was measured at 450 nm using a microplate reader (Rosys Anthos 2010, Dynex Technologies, Inc., Chantilly, VA, USA). The percentage of hemolysis was calculated relative to the positive control. The assay determines the ability of compounds to disrupt erythrocyte membranes, causing cellular lysis and hemoglobin release [49].

### 4.16. Cell Viability Assay (MTT Reduction)

HeLa cells were used for cytotoxicity evaluation. Cell counting was performed in a Neubauer chamber after trypan blue (0.4% *w*/*v*) staining. Cell suspensions were adjusted according to the equation N cells mL^−1^ × V_1_ = 1 × 10^6^ mL^−1^ to reach the desired concentration. Then, 100 µL of cell suspension was seeded into each well of a 96-well microplate (Kasvi^®^, Paraná, Brazil) and incubated for 48 h at 28 °C. After cell adhesion, the treatments were applied using 5 mg of curcuminoid film and 5 mg of curcumin film (in duplicate). The positive control consisted of 100 µL of cell suspension plus 100 µL of DMSO, and the negative control contained only the cell suspension. Plates were incubated for 24 h at 37 °C under gentle shaking (~20–30 rpm).

After incubation, the supernatant was removed, and 100 µL of MTT solution (5 mg/mL in PBS; Invitrogen, Waltham, MA, USA) was added to each well. Plates were covered with aluminum foil to prevent light exposure and incubated for 4 h at 37 °C under gentle agitation. Subsequently, 100 µL of DMSO was added to each well to solubilize formazan crystals, and absorbance was measured at 570 nm (reference 630 nm) using a microplate reader (Rosys Anthos 2010, Dynex Technologies, Inc., Chantilly, VA, USA). Metabolically active cells reduce MTT via NAD(P)H-dependent oxidoreductases to insoluble purple formazan, whose intensity correlates with cell viability [48].

### 4.17. Statistical Analysis

All experiments were performed at least in duplicate or triplicate, and the results are expressed as mean ± standard deviation (SD). Statistical analyses were carried out using GraphPad Prism version 10.0 (GraphPad Software, San Diego, CA, USA) and JASP version 0.18.3 (University of Amsterdam, The Netherlands). The normality of data distribution was verified using the Shapiro–Wilk test. For comparison among multiple groups, one-way analysis of variance (ANOVA) followed by Tukey’s post hoc test was applied. When data did not meet parametric assumptions, the Kruskal–Wallis test followed by Dunn’s multiple comparison test was employed. Differences were considered statistically significant at *p* < 0.05. Correlations between experimental parameters (e.g., viscosity, swelling, and release rate) were analyzed using Pearson’s correlation coefficient for normally distributed data or Spearman’s rank correlation for nonparametric distributions. Kinetic modeling and curve fitting for release profiles were performed by non-linear regression using least-squares minimization.

## Figures and Tables

**Table 1:** Thermal degradation parameters obtained by thermogravimetric analysis (TGA) for pure carrageenan and biofilms with or without incorporated CUR and CN56.

Sample	Td_5%_^a^ (°C)	Td_1_^b^ (°C)	Td_2_^c^ (°C)	Td_3_^d^ (°C)	T_1_^e^ (°C)	Mass Loss (%)
Pure carragenaan	56	167	185	-	-	42 *
PVA/CAR	239	256	279	448	513	91
PVA/CAR@CUR	252	277	373	449	520	92
PVA/CAR@CN56	241	271	259	448	520	91

^a^ 5% mass decomposition temperature; ^b^ Maximum decomposition temperature of the first stage; ^c^ Maximum decomposition temperature of the second stage; ^d^ Maximum decomposition temperature of the third stage; ^e^ Final decomposition temperature; * Mass loss associated with Td_1_ and Td_2_.

**Table 2:** Minimum inhibitory concentration (MIC) and minimum bactericidal concentration (MBC) values of curcumin and synthetic curcuminoids against *Gardnerella vaginalis* and *Candida albicans*.

Compound	Microorganism	Strain	MIC (µg/µL)	MBC (µg/µL)
CUR	*G. vaginalis*	ATCC 14018	0.3125	0.3125
CN56	*G. vaginalis*	ATCC 14018	10.00	10.00
CUR	*C. albicans*	Clinical isolate	0.6250	0.6250
CN56	*C. albicans*	Clinical isolate	5.00	5.00

## Data Availability

The original contributions presented in this study are included in the article. Further inquiries can be directed to the corresponding authors.

## Abbreviations

The following abbreviations are used in this manuscript:

CAR	Carrageenan
CUR	Curcumin
CN56	Synthetic curcuminoid CN56
β-CD	Beta-cyclodextrin
PVA	Poly(vinyl alcohol)
FTIR	Fourier-Transform Infrared Spectroscopy
TGA	Thermogravimetric Analysis
DSC	Differential Scanning Calorimetry
XRD	X-Ray Diffraction
SEM	Scanning Electron Microscopy
UV-Vis	Ultraviolet–Visible Spectroscopy
LE	Loading Efficiency
LDDS	Local Drug Delivery System
PBS	Phosphate-Buffered Saline
RT	Room Temperature
